# The Ultrasonic Microsurgical Anatomical Comparative Study of the CHD Fetuses and Their Clinical Significance

**DOI:** 10.1155/2015/520394

**Published:** 2015-11-10

**Authors:** Xiaosong Li, Hongmei Xia, Dan Wang, Junke Zhu, Jianhua Ran

**Affiliations:** ^1^Chongqing Center for Clinical Laboratory, Chongqing Municipal People's Hospital, Chongqing, China; ^2^Department of Anatomy and Neuroscience Center, College of Basic Medicine, Chongqing Medical University, Chongqing 400016, China; ^3^Ultrasonic Department, Affiliated Second Hospital, Third Military Medical University, Chongqing 400016, China; ^4^Key Laboratory of Molecular Biology on Infection Diseases, Chongqing Medical University, Chongqing 400016, China

## Abstract

The aim of our study was to increase the detection rate of fetal cardiac malformations for congenital heart disease (CHD). The ultrasonic and microanatomical methods were combined to study the CHD cases firstly, which could provide the microsurgical anatomical basis to the prenatal ultrasonic diagnosis which was used in suspected CHD and help the sonographer to improve the quality of fetal cardiac diagnosis. We established the ultrasonic standard section of the 175 complex CHD cases and collected the fetal echocardiography image files. The induced/aborted fetuses were fixed by 4% paraformaldehyde and dissected by the ultrasonic microsurgical anatomy. This research could obtain the fetal cardiac anatomic cross-sectional images which was consistent with the ultrasonic standard section and could clearly show the internal structure of the vascular malformation that optimized the ultrasound examination individually. This method could directly display the variation of the CHD fetal heart clearly and comprehensively help us to understand the complex fetal cardiac malformation from the internal structure of the vascular malformation which was consolidated by the anatomical basis of the fetal heart. This study could improve the integrity and accuracy of the prenatal cardiac ultrasound examination tremendously.

## 1. Introduction

Birth defects have gradually become one of the major public health problems globally and have been the main causes of infant mortality and adult disability [1]. The leading death cause of birth defects is congenital heart disease (CHD) [2–5]. CHD caused by fetal cardiovascular dysplasia is the most common congenital malformation, accompanied with developmental retardation along children growth, and leads to a great burden for the families and the whole society.

The incidence of CHD is high in China (it is reported that 0.8% to 1.2% of newborn babies have CHD). In other words, there are 200,000 CHD newborns each year [6], which tremendously restricts the improvement of population quality. Moreover, the ultrasound examination is almost the only option for prenatal diagnosis of fetal cardiac malformations so the CHD is a serious health issue with low detection rate and high morbidity, mortality, and disability rate, which causes a huge economic loss for the families and the whole society as well. The obtaining and understanding of the normal standard section are the most essential condition for abnormal fetal heart diagnosis. However, there are still some difficulties for the sonographers to diagnose the atypical congenital heart disease in clinical practice. And at present there is no systematic report about the fetal cardiac anatomy specimens which is consistent with ultrasonic standard section. Therefore, in order to improve the atypical cases diagnosis rate and prepotency, CHD cases were provided by agreement with family members and then the microanatomy and ultrasonography of the CHD cases were studied, which was a benefit to understand the complex fetal cardiac malformation comprehensively from the internal structure of the heart malformation, consolidate the anatomical basis of the fetal heart, and improve the integrity and accuracy of prenatal cardiac ultrasonic examination. This study will promote the rapid, accurate, and personalized development of fetal echocardiography.

## 2. Materials and Methods

These methods were carried out in accordance with the approved guidelines. This work was approved by the Ethical and Protocol Review Committee of the University of Chongqing Medical School (The Ethics Committee of Chongqing Medical University: 201024, 8 March 2010).

### 2.1. Materials

This study included 175 cases of induced or aborted fetus due to complex cardiac malformation at the Department of Obstetrics, the Affiliated Second Hospital of the Third Military Medical University, from January 2009 to December 2014, with gestational age 20~39 weeks (83 male cases, 92 female cases) (all specimens were provided by agreement with family members by authorized signature and the hospital ethics committee argument). The Multidimensional Color Doppler Ultrasonic Diagnostic Instrument (GE ViVid 7: the probe frequency was 2.0~4.3 MHz) was used to acquire the ultrasonic image. 4% paraformaldehyde was used to fix the CHD fetus. The Autopsy Table, Microscope, Microsurgical Scissors, Scalpel, and Forceps were used to dissect the CHD fetus. The Canon PowerShot (G6) was used to acquire the static physical image. The Vernier Caliper (measuring range: 0~150 mm, resolution: 0.02 mm) was used to measure the fetus.

### 2.2. Radiographic Study (Image Acquisition)

The standard section of fetus with complex congenital heart disease was established and the fetal echocardiography image files of the 175 cases were collected, which mainly include the apex four-chamber section (learning about the venous drainage, atrium position, atrioventricular connection order, proportion of four cavities, atrioventricular valve and pericardium, etc.), the right ventricular outflow tract section (learning about the origin and course of pulmonary artery, ventricular septum continuity), the left ventricular outflow tract section (learning about the aortic origin and course, ventricular septum continuity), the long axis of the aortic arch section (learning about the aortic isthmus), the superior mediastinum three-vessel section (learning about the position of aorta, pulmonary artery, and superior vena cava), and the long axis of fetal cardiac apex section (diagnosing the single ventricle and learning about the origin and course of the great vessels). The fetal echocardiography was examined before induced abortion, and the shape and structure of each section were observed. The diameters of each chamber and the great vessels were measured and recorded, and the image was stored which could be used for the following study [7].

### 2.3. Gross Anatomy of the Induced or Aborted Fetuses (Specimen Acquisition)

The processing and production of the specimen (4% paraformaldehyde fixed) can be outlined as follows: open the chest, remove the heart freely and record the diameter of the blood vessels and cardiac chambers, cut the heart along the ultrasonic standard section according to the characteristics of each specimen, grab image from different angle of the specimen, and put it in customized Plexiglass cylinder for archive retention. The apex four-chamber sectional anatomy is as follows: parallel to the long axis of the heart, coronal incision from the apex to the bottom of the heart, reserving the right and left atrium, the right and left ventricles, the superior and inferior vena cava, the oval foramen, valve, and partial pulmonary vein. The right ventricular outflow tract sectional anatomy is as follows: perpendicular to the long axis of heart, incision at the bottom of the heart, reserving the right ventricular outflow tract, the pulmonary artery, and the aorta. The left ventricular outflow tract sectional anatomy is as follows: starting with the pulmonary conus, making the transverse shaft parallel to the long axis of the heart, making an incision upward through the ascending aorta to the superior vena cava, down through the anterior interventricular groove to the apex, reserving partial ascending aorta, the left ventricular outflow tract, and the left ventricle. The long axis of aortic arch sectional anatomy is as follows: incision along the top of aortic arch, making the transverse shaft parallel to the long axis of aortic arch, the vertical axis point at the left atrium, exposing the aorta ascendens, arch, and descendens. The superior mediastinum three-vessel sectional anatomy is as follows: parallel to the long axis of fetal heart, making a sagittal incision through the apex to the bottom, reserving the pulmonary artery, aorta, and partial superior vena cava. The long axis of cardiac apex sectional anatomy is as follows: parallel to the long axis of fetal heart, making a coronal incision through the apex to the bottom, reserving the atrium, ventricle, or the internal structure of heart according to the location of lesions.

### 2.4. Statistical Analysis

The data were expressed as mean ± standard deviation (at least three independent experiments repetitively). Statistical analysis was performed by using one-way analysis of variance (ANOVA) which was used to assess the differences between groups, and the laboratory data were considered to be statistically significant when *P* < 0.05. All data were processed by the Statistical Package for the Social Sciences statistical software for Windows, version 19.0.1 (SPSS, Inc., Chicago, IL, USA).

## 3. Results

### 3.1. 175 Cases of Fetal Complex Cardiac Malformation Anatomical Specimens and Corresponding Echocardiographic Image Data Were Successfully Acquired in This Research

**Figure 1:**
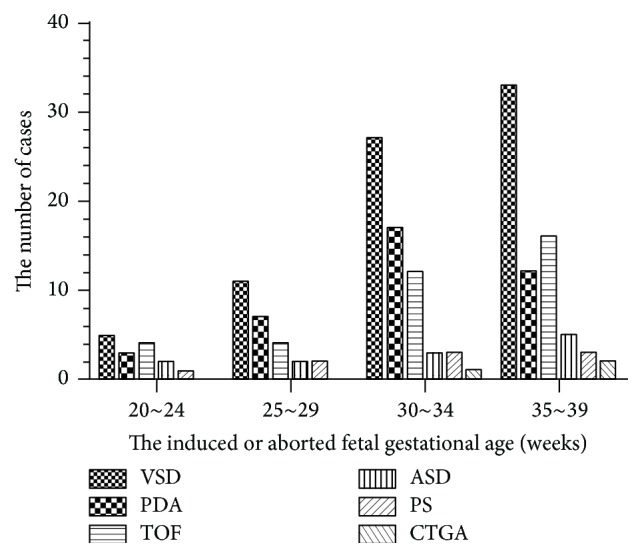
The variation type and cases of the induced or aborted fetus from 2009 to 2014. VSD: ventricular septal defect; PDA: patent ductus arteriosus; TOF: Tetralogy of Fallot; ASD: atrial septal defect; PS: pulmonary stenosis; CTGA: complete transposition of the great arteries.

Cases included 76 cases of ventricular septal defect (VSD), 39 cases of patent ductus arteriosus (PDA), 36 cases of Tetralogy of Fallot (TOF), 12 cases of atrial septal defect (ASD), 9 cases of pulmonary stenosis (PS), and 3 cases of complete transposition of the great arteries (CTGA), as shown in Figure 1 and Table 1. Generally speaking, certain regularity could be observed inside and outside the right atrium which was positively correlated with gestational age; the altered growth and development of the left atrium was the same as the right atrium. The size and extent of variation in ventricular septal defect, patent ductus arteriosus, Tetralogy of Fallot, atrial septal defect, pulmonary stenosis, complete transposition of the great arteries, aorta, aorta velocity, pulmonary artery, and pulmonary artery velocity presented irregular change with gestational age. The ventricular septal defect was the most common cardiac anomaly which was similar to the one ever reported [8, 9].

### 3.2. The Induced or Aborted Fetal Echocardiography Measurements

175 cases of fetal complex cardiac malformation anatomical specimens at 20–39 weeks of gestation were prospectively evaluated. As shown in Table 1, the internal diameter of pulmonary artery (PA) and aorta (AO) showed positive correlation with the increasing of gestational age (*P* < 0.05), but the PA/AO ratio remained comparatively steady (1.1–1.2). The blood velocity in aorta and pulmonary artery remained steady along with the gestational age (*P* > 0.05). The internal diameter of PA and AO and the PA/AO ratio are the most valuable diagnosis in primary screening of CHD, especially for large artery abnormities.

### 3.3. The Apex Four-Chamber Echocardiography Section and the Corresponding Sectional Anatomy

**Figure 2:**
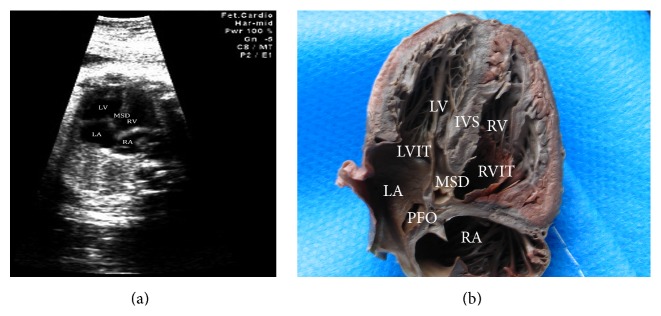
Ventricular septal defect (VSD) (32 w). (a) The apex four-chamber echocardiography section. (b) The apex four-chamber sectional anatomy. LA: left atrium; LV: left ventricle; RA: right atrium; RV: right ventricle; PFO: patent foramen ovale; IVS: interventricular septum; LVIT: left ventricular inflow tract; RVIT: right ventricular inflow tract; MSD: membranous septal defect.

The section is one of the basic sections of fetal echocardiography. We can identify the left atrium, right atrium, left ventricle, and right ventricle according to CHD cases heart's specifically anatomical characteristics. For example, the left atrium has the pulmonary vein drainage and its inner wall is smooth. The right atrium has the superior and inferior vena cava drainage; the coronary sinus openings and the pectinate muscles in right atrium are clear. For the left ventricle, the endocardium was smooth and complete, and the apex is smoother and thinner than the right ventricle. For the right ventricle, the apex is full as there are many trabeculations and the inner wall is coarser. There is an image of VSD (diameter of 2.8 millimeters); the fetal right atrium is bigger than left atrium and the right ventricle is bigger than left ventricle obviously (Figure 2).

### 3.4. The Right Ventricular Outflow Tract Echocardiography Section and the Corresponding Sectional Anatomy

**Figure 3:**
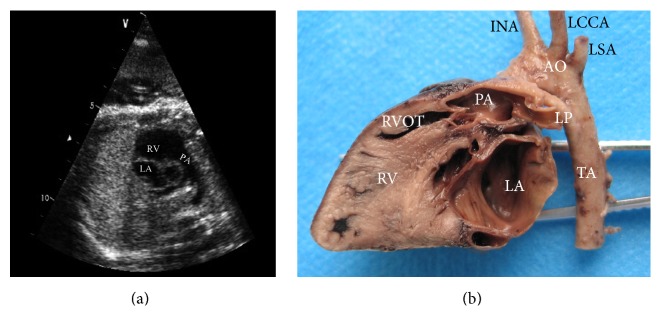
Tetralogy of Fallot (TOF) (28 w). (a) The right ventricular outflow tract echocardiography section. (b) The right ventricular outflow tract sectional anatomy. INA: innominate artery; LCCA: left common carotid artery; LSA: left subclavian artery; PA: pulmonary artery; RVOT: right ventricular outflow tract; RV: right ventricle; LA: left atrium; AO: aorta; LPA: left pulmonary artery; TA: thoracic aorta.

The right ventricular outflow tract sectional anatomy shows the right ventricle, right ventricular outflow tract, pulmonary artery, pulmonary valve, and pulmonary arterial branch. In this echocardiography section, we can observe the development and function of tricuspid valve and pulmonary valve, evaluating the valvular regurgitation, stenosis of outflow tract, and pulmonary artery and branches. The section showed higher rates for diagnosis to the Tetralogy of Fallot and/or merged with pulmonary stenosis, which could evaluate the surgical postoperative effect after birth effectively. There is an image of TOF (Figure 3).

### 3.5. The Left Ventricular Outflow Tract Echocardiography Section and the Corresponding Sectional Anatomy

**Figure 4:**
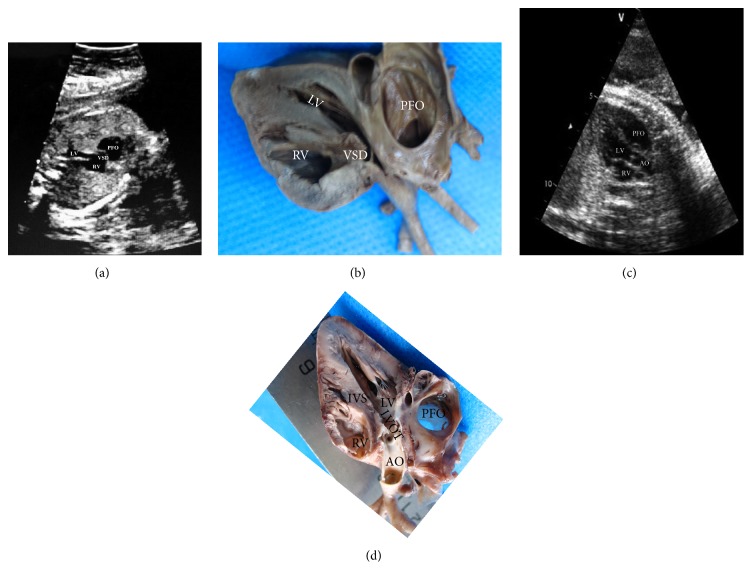
Atrial septal defect (ASD) (37 w). (a) The left ventricular outflow tract echocardiography section. (b) The left ventricular outflow tract sectional anatomy. Aortic stenosis (AS) (39 w). (c) The left ventricular outflow tract echocardiography section. (d) The left ventricular outflow tract sectional anatomy. LV: left ventricle; RV: right ventricle; PFO: patent foramen ovale; AO: aorta; IVS: interventricular septum; LVOT: left ventricular outflow tract; PA: pulmonary artery.

The left ventricular outflow tract sectional anatomy could show the anteroposterior diameter of the ventricular and internal structure clearly, which helps to diagnose the congenital heart disease such as aorta overriding, Atrial septal defect (ASD), and aortic stenosis (AS). The images of ASD (the area is 3.20 cm^2^) and AS (the diameter is 2.80 mm) are given in Figure 4.

### 3.6. The Long Axis of Aortic Arch Echocardiography Section and the Corresponding Sectional Anatomy

**Figure 5:**
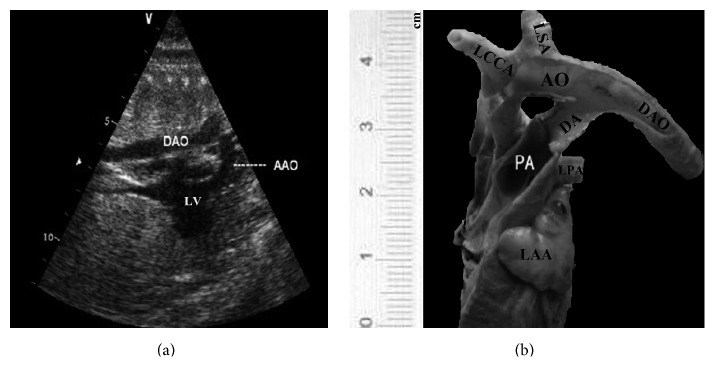
Patent ductus arteriosus (PDA) (38 w). (a) The long axis of aortic arch echocardiography section. (b) The long axis of aortic arch sectional anatomy. AAO: ascending aorta; LV: left ventricle; AO: aorta; DA: arterial duct; DAO: descending aorta, PA: pulmonary artery; LPA: left pulmonary artery; LCCA: left common carotid artery; LSA: left subclavian artery; LAA: left atrial appendage.

This section showed higher rates for diagnosis to the Tetralogy of Fallot and/or merger pulmonary stenosis, which could evaluate the surgical postoperative effect after birth effectively. The shape of the aortic arch was similar to “walking stick”; its curvature was bigger and has three branches such as brachiocephalic trunk, left common carotid artery, and left subclavian artery. The ductus arteriosus between the descending aorta and left pulmonary artery was an important channel of the circulatory system while in the womb, which would close within 24 to 48 hours after birth usually. The infant would be considered to be CHD (patent ductus arteriosus, PDA) if it is still not closed after a few weeks. This section could help to diagnose the patent ductus arteriosus (PDA). The image of PDA (the diameter was 7.10 mm) is given in Figure 5. By measurement, the diameter of the pulmonary artery is 8.10 mm, the aorta is 7.80 mm, and the ductus is 7.10 mm, which has an abnormal proportion.

### 3.7. The Superior Mediastinum Three-Vessel Echocardiography Section and the Corresponding Sectional Anatomy

**Figure 6:**
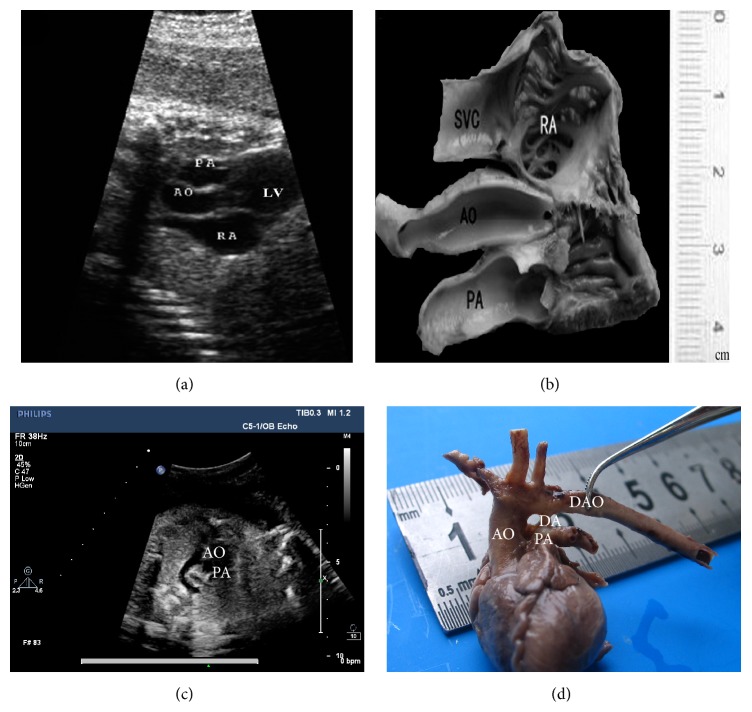
Aortic stenosis (AS) (38 w). (a) The superior mediastinum three-vessel echocardiography section. (b) The superior mediastinum three-vessel sectional anatomy. Pulmonary artery hypoplasia (PAH) (30 w). (c) The superior mediastinum three-vessel echocardiography section. (d) The superior mediastinum three-vessel sectional anatomy. SVC: superior vena cava; AO: Aorta; PA: Pulmonary Artery; RA: Right Atrium; LV: left ventricle; DAO: descending aorta; DA: ductus arteriosus.

The section includes the superior vena cava, aorta, pulmonary artery, and right atrium, and they are parallel normally. The aorta is in the middle, and the pulmonary artery and superior vena cava are at both sides of the aorta. Among the three vessels, the diameter of pulmonary artery is greater than aorta and aorta is greater than superior vena cava. This view is an important section of screening fetal vascular malformation; there is an image of AS (the diameter is 3.80 mm) given in Figure 6.

### 3.8. The Long Axis of Cardiac Apex Echocardiography Section and the Corresponding Sectional Anatomy

**Figure 7:**
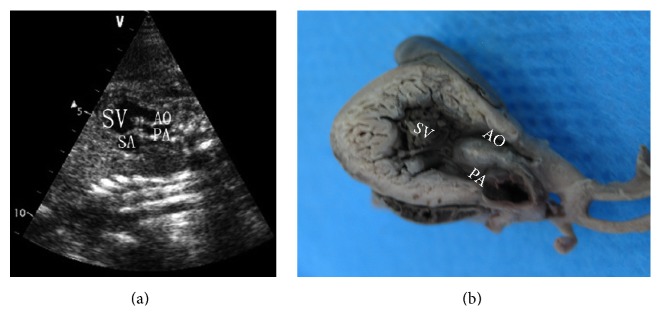
Single ventricle (SV) (24 w). (a) The long axis of cardiac apex echocardiography section. (b) The long axis of cardiac apex sectional anatomy. SV: single ventricle; AO: aorta; PA: pulmonary artery; SA: single atrium.

This view is an important section of diagnosing the single ventricle and the origin and course of the great vessels. Under normal circumstances in this section, the structure of the left ventricular myocardium was smooth and dense; the right ventricular trabecula was rich and compact, and the spatial structure between the aorta and pulmonary artery was normal and had parallel relationship. For example, the ventricles have only one cavity; the myocardium is loose and hypertrophic. The courses of pulmonary artery and aorta are parallel and open in the single ventricular cavity together. There is an image of SV (Figure 7).

## 4. Discussion

In order to detect abnormal process, the sonographer must strictly confirm the cardiac ultrasonic examination protocols and be familiar with the standard section. Now there is no systematic report about the fetal cardiac anatomy specimen which is consistent with ultrasonic standard section. With the rapid development of digital medicine, digitized visible human organs play an important role in the reconstruction of three-dimensional image, navigation of surgical and interventional procedures, preoperative virtual simulation, and the telemedicine. The preparation and digital three-dimensional reconstruction of fetal cardiac anatomy specimens will be of benefit for comprehensively understanding the complex fetal cardiac malformation from the internal structure of the vascular malformation, consolidating the anatomical basis of the fetal heart, improving the integrity, efficiency, timeliness, and accuracy of prenatal cardiac ultrasound examination. This study will promote a rapid, accurate, and personalized development of fetal echocardiography.

The incidence of CHD in live birth infants is 8‰~11‰, and the death toll in perinatal and neonatal infants accounts for about 50% of all deaths during this period. CHD is one of the deadly diseases of high incidence and high mortality, which is a major worldwide health problem, and the ASD is the most frequent type of congenital heart disease [10]. Furthermore, the overall mortality rate of congenital heart disease (CHD) has increased in China [11, 12]. As previously reported, the parental subfertility was associated with an increase in abdominal wall defects, penoscrotal hypospadia, right ventricular outflow tract obstruction, and methylation defects causing imprinting disorders [13]. The latest research has reported the MTHFR-677T allele as a susceptibility factor for CHD in the Asian maternal population and the −1298C allele as a risk factor in the Caucasian paediatric population [14]. Therefore, mastering the anatomy of the complex fetal cardiac malformation and the echocardiographic images is very important for the prenatal eugenics choice and postnatal timely treatment. However, the fetus echocardiography is the key point of prenatal examination, whose sensitivity and accuracy are restricted by the inspectors' technical level. The main reason is that the rareness, abstractness, and complexity of fetal cardiac malformations often interfere with the diagnosis, which requires the sonographers to have the ability to identify the inner anatomic structure of fetal heart and to have a very strong operating skill. The acquisition and understanding of fetal echocardiography standard sections are the most basic condition to find the fetal cardiac abnormality; therefore, the sonographer must strictly confirm the cardiac ultrasound examination protocols and be familiar with the standard sections. The preparation and digital three-dimensional reconstruction of fetal cardiac anatomy specimens will be of benefit for comprehensively understanding the complex fetal cardiac malformation from the internal structure to the vascular malformation, consolidating the anatomical basis of the fetal heart, improving the integrity, fasting, and accuracy of prenatal cardiac ultrasound examination. The prenatal diagnosis of CHD is about 57% by using the apex four-chamber section, which has an important value of diagnosing the left heart, right heart, and tricuspid dysplasia, mitral stenosis or atresia, ventricular septal defect, single ventricle, single atrium, and pericardial effusion [15–20]. The right ventricular outflow tract echocardiography section is an important section of diagnosing the tricuspid valve, right ventricular outflow tract, pulmonary artery, and pulmonary valve malformation, such as the right ventricular outflow tract stenosis and pulmonary valve malformation, while the left ventricular outflow tract echocardiography section is an important section of diagnosing the left ventricular outflow tract, aorta, and aortic malformation, such as the left ventricular outflow tract obstruction, ventricular septal defect, aortic valve abnormalities, and left heart dysplasia. The study focused on the microdissection of complex fetus cardiac malformation in different anatomical plane according to different malformation, showing the specific internal structure such as single ventricle, double outlet of right ventricle, left ventricular dysplasia, TOF, CTGA, and pulmonary atresia. Comparing with the corresponding echocardiography sections, the ultrasonic image characteristics of complex congenital heart disease should be understood visually, further ensuring the quality of examination by different inspectors, reducing the rate of misdiagnosis and missed diagnosis. With the rapid development of the ultrasonic technology, prenatal ultrasound examination has become the most commonly used fetal prenatal screening, with its advantage of being noninvasive, repeatable, and reliable. When abnormal fetal development is shown, it can provide direct basis for the clinical early intrauterine intervention. Meanwhile, studies have shown that the use of ultrasonography to guide first-trimester pregnancy terminations has obviated the need for sharp curettage and to screen for external ear abnormality in the fetuses [21, 22]. However, the basic education of fetal anatomy is lacking. In the clinical practice, prenatal echocardiography is often used to diagnose the fetal cardiac malformation and evaluate the cardiac function. The two-dimensional ultrasound can accurately diagnose all kinds of cardiac malformation, but there are some limitations in the evaluation of cardiac function, while the real-time three-dimensional echocardiography (RT-3DE) can avoid the disadvantages of single detection in two-dimensional section and evaluate the heart function comprehensively [23]. However, only when the fetal heart is large is the image resolution of RT-3DE clear. What is more, some images are clear with the two-dimensional (2D) echocardiography but could not be gotten with RT-3DE. So the image resolution of RT-3DE needs to be improved [24–26]. In recent years, some new technology in fetal heart examination such as 3D and 4D ultrasound has become a hot research field and has gained a lot of new progress, especially the Spatiotemporal Image Correlation (STIC) which opens up a new window for fetal heart examination and provides the morphological basis for intrauterine therapy of CHD at the same time [27–30]. It was reported that the 64-detector computed tomography could provide a good diagnostic performance in congenital heart disease [31], which depends on the understanding of normal fetal cardiac anatomic structure; meanwhile, some new studies demonstrated that the identification and characterization of the cellular and molecular pathways involved in the differentiation and morphogenesis of specific cell types of the developing heart were crucial to understanding the process of cardiac development and the pathology associated with human congenital heart disease, and they found that the CHD5 (congenital heart disease 5 protein) was essential for CASZ1 (cardiac transcription factor CASTOR) function and that the CHD5-CASZ1 interaction was necessary for cardiac morphogenesis and numerous signaling pathways were critical for normal valve development which could be reexpressed in diseased valves [32–35]. The increased level of PTX3 is also an independent predictor of combined end point of left ventricle dysfunction or mortality at one year; combining the biomarker and the device could be of great utility since they monitor the severity of two pathophysiological different mechanisms: heart fibrosis and fluid overload [36]. However, the data of fetal heart gross anatomy is rare. Due to the influence of gestational age, fetal position, fetal movement, sampling volume position, the image resolution of echocardiography, and so forth, the echocardiography of fetal cardiac morphology is often less precise, and it is rarely reported to measure the fetal heart directly by the ultrasonic microdissection methods at home and abroad. Therefore, understanding the features of fetal heart development and morphology at different periods has an important reference value for the understanding, diagnosis, and surgical therapy of fetal cardiac malformation. The frequent association of fetal CHD and chromosomal and extracardiac pathology emphasises the importance of thorough evaluation of any fetus with CHD [37]. Recently, some scholars [38] have reported that CHD are rarely caused by mutations in cardiac and smooth muscle actin genes. The genetic factors may have important roles in the development of CHD, and TOF and VSD may have similar molecular mechanisms [39]. But other scholars [40] have reported that there were some novel mutations in the transcriptional activator domain of the human TBX20 with ASD.

The fetal anatomy section and the corresponding ultrasound standard section in the systematic ultrasound examination of fetus (20–39 weeks) were obtained in this study, which could provide technical guidance for clinical diagnosis of CHD and reduce the rate of missed diagnosis. What is more, this study could also provide more intuitive, reliable, and convenient reference of fetal heart variation for sonographers. Thus, each ultrasonic diagnostic standard section of fetal cardiac malformations could be rapidly scanned and the correlation between the main structure of the fetal heart and great vessels could be comprehensively evaluated in a short time. Furthermore, different inspectors could obtain the same ultrasonic diagnostic quality as much as possible, which could reduce the missed diagnosis, raise the sensitivity and accuracy of fetal echocardiography, improve population quality, and lessen the mortality of the low age.

This study may help sonographers to provide more favorable three-dimensional images so that they can better serve the community. This was the first comparative study to ever report such an association between the ultrasonic diagnosis and anatomical research on the CHD fetus. However, due to the restraints on resources and time, this research is subject to improvement on index selection, sample size, and methodology; we will increase the sample size reasonably and improve the research methods in the later experiment which could provide a real-time dynamic variation of three-dimensional images for clinical accurate diagnosis. In the next trial, we will collect more typical cases and imaging data of the fetal heart. After being fixed in 10% formalin and going through 64 lines of CT scanning, the heart of the CHD has been embedded in 5% of green gelatin and put into the −30°C icehouse for a week. Cut it from top to bottom layer by layer with a digital milling machine TK-6350 in −25°C laboratory. Take pictures of the fetus layer by layer with a high definition digital camera and obtain the fetus data and structural database. Then, reconstruct the 3D image of the anatomic structure of the CHD hearts, using software named Amira.

## Figures and Tables

**Table 1:** The induced or aborted fetal echocardiography measurements ($\overset{-}{x}\pm s$) (*P* < 0.05).

Week(s)	VSD	PDA	TOF	ASD	PS	CTGA	PA (mm)	AO (mm)	PA/AO	PAV (cm/sec)	AOV (cm/sec)
20~24 w	5	3	4	2	1		5.87 ± 0.56	4.85 ± 0.91	1.21	70.12 ± 10.21	81.34 ± 10.53
25~29 w	11	7	4	2	2		6.76 ± 0.33	5.69 ± 0.87	1.18	73.42 ± 11.91	84.46 ± 11.85
30~34 w	27	17	12	3	3	1	7.24 ± 0.78	6.54 ± 1.28	1.11	76.95 ± 11.57	88.72 ± 12.52
35~39 w	33	12	16	5	3	2	8.56 ± 1.12	7.58 ± 1.86	1.13	81.59 ± 12.32	93.87 ± 13.75

Most common cardiac diagnosis of infants undergoing cardiac surgery from 2009 to 2014. VSD: ventricular septal defect; PDA: patent ductus arteriosus; TOF: Tetralogy of Fallot; ASD: atrial septal defect; PS: pulmonary stenosis; CTGA: complete transposition of the great arteries; AO: aorta; AOV: aorta velocity; PA: pulmonary artery; PAV: pulmonary artery velocity. The laboratory data were considered to be statistically significant when *P* < 0.05.

## Abbreviations

VSD: Ventricular septal defect
PDA: Patent ductus arteriosus
TOF: Tetralogy of Fallot
ASD: Atrial septal defect
PS: Pulmonary stenosis
CTGA: Complete transposition of the great arteries
AO: Aorta
AOV: Aorta velocity
PA: Pulmonary artery
PAV: Pulmonary artery velocity.
